# Changes in Higher-Order Chromosomal Structure of *Klebsiella pneumoniae* Under Simulated Microgravity

**DOI:** 10.3389/fmicb.2022.879321

**Published:** 2022-05-30

**Authors:** Yahao Wang, Wenlong Shen, Man Yin, Wenhua Huang, Bingyu Ye, Ping Li, Shu Shi, Ge Bai, Xinjie Guo, Yifei Jin, Kailin Lin, Yan Zhang, Yongqiang Jiang, Junfeng Wang, Yanping Han, Zhihu Zhao

**Affiliations:** ^1^Beijing Institute of Biotechnology, Beijing, China; ^2^Institute of Genetics and Developmental Biology, Chinese Academy of Sciences, Beijing, China; ^3^State Key Laboratory of Pathogen and Biosecurity, Beijing Institute of Microbiology and Epidemiology, Beijing, China; ^4^College of Life Science, Henan Normal University, Xinxiang, China; ^5^Second Medical Center of Chinese People’s Liberation Army (PLA) General Hospital, Beijing, China

**Keywords:** simulated microgravity, Hi-C, chromosome conformation, transcriptome, methylation

## Abstract

Our previous work have shown that certain subpopulations of *Klebsiella pneumoniae* exhibit significant phenotypic changes under simulated microgravity (SMG), including enhanced biofilm formation and cellulose synthesis, which may be evoked by changes in gene expression patterns. It is well known that prokaryotic cells genomic DNA can be hierarchically organized into different higher-order three-dimensional structures, which can highly influence gene expression. It is remain elusive whether phenotypic changes induced by SMG in the subpopulations of *K. pneumoniae* are driven by genome higher-order structural changes. Here, we investigated the above-mentioned issue using the wild-type (WT) *K. pneumoniae* (WT was used as a control strain and continuously cultivated for 2 weeks under standard culture conditions of normal gravity) and two previous identified subpopulations (M1 and M2) obtained after 2 weeks of continuous incubation in a SMG device. By the combination of genome-wide chromosome conformation capture (Hi-C), RNA-seq and whole-genome methylation (WGS) analyses, we found that the along with the global chromosome interactions change, the compacting extent of M1, M2 subpopulations were much looser under SMG and even with an increase in active, open chromosome regions. In addition, transcriptome data showed that most differentially expressed genes (DEGs) were upregulated, whereas a few DEGs were downregulated in M1 and M2. The functions of both types DEGs were mainly associated with membrane fractions. Additionally, WGS analysis revealed that methylation levels were lower in M1 and M2. Using combined analysis of multi-omics data, we discovered that most upregulated DEGs were significantly enriched in the boundary regions of the variable chromosomal interaction domains (CIDs), in which genes regulating biofilm formation were mainly located. These results suggest that *K. pneumoniae* may regulate gene expression patterns through DNA methylation and changes in genome structure, thus resulting in new phenotypes in response to altered gravity.

## Introduction

Outer space is a unique environment, and microgravity is one of the key factors that can induce genetic or epigenetic variations in earth-born organisms. Previous studies have mainly focused on exploring the effects of microgravity at cellular and molecular levels. For example, the biofilm thickness of *Escherichia coli* has been shown to increase in outer space (Zea et al., 2017), and numerous genes in *E. coli*, especially those related to stress and biofilm formation, undergo significant changes under simulated microgravity (SMG) (Vukanti et al., 2008). Phenotypic changes in *Candida albicans* under spaceflight are accompanied by differential expression of genes related to stress and drug resistance (Crabbé et al., 2013). Although several studies have shown that that microbial growth, biofilm formation, virulence, and drug resistance are affected under microgravity (Baker et al., 2004; Lynch et al., 2006; Wang et al., 2017; Aunins et al., 2018; Yin et al., 2020; Su et al., 2021), the specific genetic or epigenetic mechanisms by which the space environment or SMG affects microorganisms remain unknown. In our previous study, a strain of *Vibrio natriegens* with a slower growth rate was obtained under SMG, which may be caused by changes in its chromosomal structure. *V. natriegens* has two chromosomes, and there existed extensive interaction between the ori region of Chr. 2 and the crtS site of Chr.1 to coordinate the replication process of chromosomes. Under SMG, the interaction frequency of this region decayed significantly, and also there was a reduced interaction frequency in the replication terminus region of the Chr. 2. These indicated that the change of chromosome structure at least part of the reason for slower growth phenotype observed (Yin et al., 2020). Moreover, it suggested that higher-order structure of chromosome may play a key role in microbial adaptation to microgravity environments. However, studies on the effect of altered gravity on the microbial chromosomal structures are limited.

Similar to eukaryotic genomic DNA, bacterial genomic DNA is hierarchically folded and compressed by nuclear-associated structural and other proteins. The functional units of chromosomes with different hierarchical structures are further driven by DNA supercoiling and transcription status (Le et al., 2013; Wang et al., 2013; Le and Laub, 2016; Qin et al., 2019; Verma et al., 2019; Lourenço et al., 2020). Genome-wide chromosome conformation capture (Hi-C) assays are the robust technique to analyze the three-dimensional (3D) conformation of chromosomes. Hi-C assays provide quantitative information on the spatial proximity of genomic loci by counting the frequency of contacts between different regions of chromatin, thus exploring the relationship between the spatial location of the entire chromatin DNA on a genome-wide scale (Lieberman-Aiden et al., 2009). Over the past decade, Hi-C assays have been widely used to unveil the 3D structure and organization of bacterial genomes. Bacterial genomes are further subdivided into chromosomal interaction domains (CIDs) with an average length of approximately 100 kb (Marbouty et al., 2015; Wang et al., 2015; Lioy et al., 2018). Genes within the same CIDs interact more frequently than those in different domains, indicating that CIDs act as fundamental structures of the bacterial genome and play important roles in various biological processes, including chromosome replication and gene expression regulation. Therefore, it is necessary to probe the chromosomal conformation of bacterial genomes to explore their changes under different conditions, such as microgravity environments.

*Klebsiella pneumoniae* is widely present in nature and is an important causative agent of hospital-acquired infections (Mohd Asri et al., 2021). This bacterium has been previously isolated from orbiting spacecrafts and post-flight astronauts (Novikova, 2004). Due to the widespread use of antimicrobials, *K. pneumoniae* has developed multidrug resistance (Mohd Asri et al., 2021). Most current studies on *K. pneumoniae* have focused on drug resistance and clinical infection treatment, and some articles have explored the pathogenic mechanism of *K. pneumoniae* under SMG or microgravity (Li et al., 2014; Wang et al., 2016). However, there is a lack of articles on genetic and epigenetic studies in *K pneumoniae* under microgravity. In our previous work (Wang et al., 2017), by incubating wild-type *K. pneumoniae* continuously for 2 weeks under SMG, we observed that growth characteristics of *K. pneumoniae* exhibited phenotypic heterogeneity. According to morphological observation, including colony morphology and bacterial phenotype, two subpopulations strains, M1 and M2 were isolated. Biofilm analysis, Quantitative real-time PCR and other methods were used to determine the features of subpopulations and related gene expression changes. Compared with WT, especially M2 has stronger biofilm formation ability. However, whole-genome resequencing analysis found that there were no convincing single nucleotide polymorphisms among WT, M1, and M2. This means that these phenotypic differences may be caused by non-genetic variations, but more evidence is needed to prove this.

In this study, we used the Hi-C assay technique to obtain the chromosomal structures of these subpopulations of *K. pneumoniae* and integrated these results with transcriptome and methylation data. Our results showed significant changes in chromosome conformation under SMG conditions, and these changes were associated with differential gene expression and DNA methylation. In conclusion, our study provides new insights into the response mechanisms of microorganisms to microgravity environments.

## Materials and Methods

### Bacterial Strains Used for the Experiments

Wild-type (WT) *K. pneumoniae* (ATCC BAA-1705), as well as its two subpopulations M1, M2 isolated from bacterial culture in simulated microgravity environment, were used in this study. WT strains were aliquoted, one as a control strain was cultured continuously for 2 weeks under the standard culture condition of normal gravity and the other was cultured in a HARV bioreactor filled with LB medium at 37°C with a rotational of 25 r.p.m, and passaged every 24 h (Wang et al., 2017). After 2 weeks of continuous incubation, the bacterial cultures were collected and transferred to LB agar plate and incubated at 37°C for 24 h under standard culture conditions. From the SMG group, M1 and M2 subpopulations were isolated according to the different morphology of the colonies. However, the colony morphology of the control group did not change compared with 2 weeks ago. Furthermore, the experiments also found that the colony morphology and bacterial cell arrangement of M1 and M2 subpopulations remain unchanged at least within five passages under the standard culture condition *in vitro*. So the control strain, M1 and M2 subpopulation strains were separately inoculated into LB medium and kept at 37°C with 200 r.p.m for 24 h under standard culture conditions. Then bacteria cultured were collected and flash-frozen in liquid nitrogen at –80°C for subsequent experiments. In our experiments, cryopreserved strains were activated under standard culture conditions and collected the bacteria cultured to the early logarithmic phase to prepare samples.

### Genome-Wide Chromosome Conformation Capture and Data Analysis

The protocol for the Hi-C assay was performed as previously described (Yin et al., 2020). Bacteria cultured to the early logarithmic phase were collected by centrifugation and washed twice with cold PBS. A final concentration of 3% fresh formaldehyde was used to crosslink the bacteria (1 × 10^9^ cells) at room temperature (RT) for 10 min. The formaldehyde was quenched by incubation with a final concentration of 0.375 M glycine at 4°C for 20 min and the sample was stored at –80°C or used directly for subsequent experiments. The cross-linked bacterial samples were resuspended in Tris-EDTA (TE), followed by addition of lysis buffer (50 U/μL). Lysis was performed at 37°C in an incubator for 10 min and followed by addition of SDS to a final concentration of 0.5% to accelerate lysis and the samples were left for a further 10 min at RT. 10% Triton X-100 was used to terminate the lysis reaction and the samples were incubated at RT for 10 min. After centrifugation, the supernatant was discarded and the DNA that collected in the pellet was digested by adding the digestion system (10 × NEB buffer 2.1 and 100 U of *Sau*3AI) and placing in an incubator at 37°C. The end-complementation system (10 × 2.0 NEB buffer, Klenow, 10 mM dATP/dTTP/dGTP, 5 mM biotin-16-AA-2′dCTP) was added and the reaction was left at RT for 4 h. Blunt-end ligation was performed using T4 DNA ligase with 10.0 mL final volume (Thermo Fisher Scientific, United States) at 16°C overnight. After ligation, RNaseA (Thermo Fisher Scientific, United States) was added to remove residual RNA and 200 mg/mL proteinase K (Thermo Fisher Scientific, United States) was added before incubation at 65°C for 5 h. DNA was purified and recovered using AMpure beads (Beckman, United States) according to the manufacturer’s protocol. The samples were sonicated for 60 s using Covaris M220 and the DNA was sheared to ∼400 bp length. Then, AMpure beads (Beckman) with an equal volume was used to purify and recover the DNA according to the manufacturer’s protocol. The target biotinylated fragments were captured using Dynabeads™ MyOne™ Streptavidin C1 (Thermo Fisher Scientific, United States). Hi-C libraries were constructed with 200 ng DNA as input amounts using the NEBNext^®^ Ultra™ II DNA Library Prep Kit for Illumina (NEB, United States) according to the manufacturer’s protocol. Fragment lengths from 400 to 600 bp were analyzed on an Illumina HiSeq X Ten platform (San Diego, CA, United States) with paired -end sequencing and two replicates per set of samples, for a total of 6 libraries.

We got about 20 million reads for each library. For the downstream data from high-throughput sequencing, we first filtered the raw data to remove unqualified reads, the data quality control were checked using MultiQC with default parameters (Ewels et al., 2016), and used bowtie2 (Langmead and Salzberg, 2012) to map the clean data to the reference genome (GCF_000240185.1) with default parameters. The resulting files were subsequently processed using HiCExplorer (Wolff et al., 2020) to generate the DNA-DNA interaction matrix files and the correlation between each sample and each replicate was analyzed. Then the two biological replicates were merged for subsequent analysis. The original Hi-C matrices were further normalized and corrected for data bias (including uneven distribution of restriction fragment lengths, ligation efficiency, and nucleotide composition) using the HiCExplorer tools. 2 kb resolution was used to identify local features of chromatin and for subsequent identification of relatively active and inactive regions of chromosomes (A/B compartments) (Takemata et al., 2019; Misteli, 2020), as well as CID analysis. Calling chromosome A/B compartments using cooltools with default parameters (Venev et al., 2021) and a script of cworld, matrix2insulation.pl (with parameters –is 100000 –ids 52000 –nt 0.2) was used to analyze CID boundaries (Lajoie and Venev, 2016).

### Transcriptome Analysis

Samples were collected by centrifugation and discarded the supernatant. The cells (approximately 5 × 10^6^) were treated with TRIzol reagent (Sigma-Aldrich, United States) and pipetted up and down until the liquid was mixed and incubated for 5 min at RT. Added 0.2 mL of chloroform per 1 mL of TRIzol Reagent used for lysis, then thoroughly mixed by shaking and incubated for 10 min at RT. Centrifuged the sample for 15 min at 12,000 × g at 4°C. Transferred the aqueous phase containing the RNA to a new tube. Added 0.5 mL of isopropanol to the aqueous phase and incubated for 20 min at RT. Centrifuged for 20 min at 12,000 × g at 4°C and discarded the supernatant. Washed RNA with 75% ethanol and discarded the supernatant by centrifugation, then air dry the RNA pellet for 5–10 min. Resuspended the pellet in 20 μL of RNase-free water by pipetting up and down. NanoPhotometer spectrophotometer^®^ (Bio-Rad, Hercules, CA, United States) was used to check the purity of RNA. Then RNA concentration was measured using Qubit^®^ RNA Assay Kit in Qubit 3.0 Fluorometer (Life Technologies, CA, United States). RNA integrity was evaluated using the RNA Nano 6000 Assay Kit of the Agilent Bioanalyzer 2100 system (Agilent Technologies, CA, United States). Next, cDNA library was constructed using NEBNext^®^ Ultra™ RNA Library Prep Kit for Illumina (NEB, United States) with 1 μg RNA as input amounts. With two replicates per sample, a total of 6 libraries were constructed. Libraries were deeply sequenced using an Illumina Hiseq X Ten platform and generated 150 bp paired-end reads.

Mapping values per kilobase of transcript per million fragments were calculated to determine the transcript expression levels. DESeq2 (Love et al., 2014) was used to analyze gene expression and the absolute value of the log2FoldChange ≥ 1 criterion was used to identify differentially expressed genes in the WT, M1, and M2 strains. GO and KEGG functional annotation analyses were performed using DAVID (Huang et al., 2009a,b) and KOBAS-I (Bu et al., 2021).

### Methylation Sequencing Analysis

Cells (about 1 × 10^9^ cells) were Collected and used the QIAamp DNA Mini Kit (Qiagen, Germany) to extracted DNA according to the manufacturer’s instructions. Then DNA concentration was measured using Qubit^®^ DNA Assay Kit in Qubit 3.0 Fluorometer (Life Technologies, CA, United States) and DNA quality was assessed by 1% agarose gel electrophoresis. Methylation libraries were constructed by using the NEBNext^®^ Enzymatic Methyl-seq Kit (NEB) with 200 ng DNA as input amounts. And referred to the manufacturer’s protocol, we prepped libraries with fragments size of ∼400 bp for each sample (no biological replicates, a total of 3 libraries). High-throughput sequencing was performed using the Illumina sequencing platform, and the sequencing read length was 150 bp paired-end reads.

We got 3.3G of raw data in total. And bismark (Krueger and Andrews, 2011) was used to identify and extract methylation sites. The R package DSS (Wu et al., 2015) was used to identify differentially methylated regions of different components, using a threshold of *P*-value < 0.005.

## Results

### Higher-Order Chromosomal Structures Became Looser in M1 and M2 Subpopulation

**FIGURE 1:**
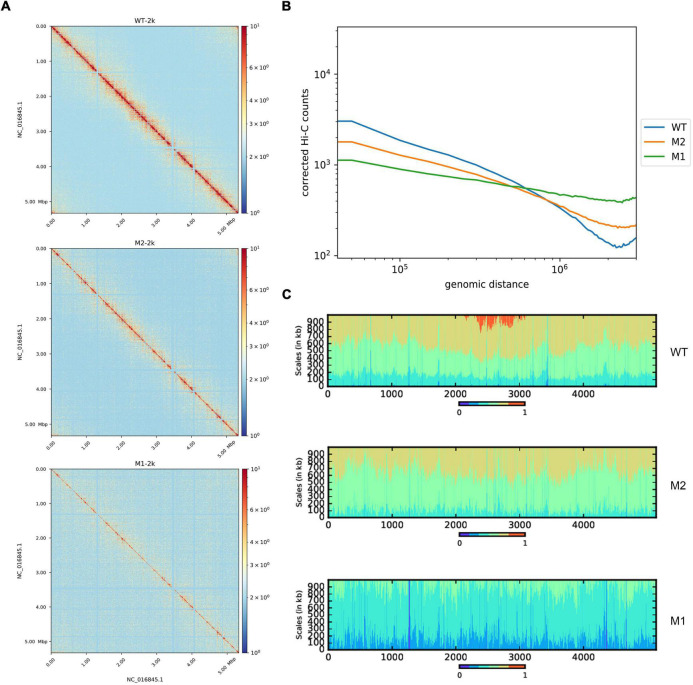
Whole-chromosome organization of WT, M1, and M2. **(A)** Chromosome contact map of WT, M1, and M2 after normalization (2 kb resolution), x and y axes represent genomic coordinates. The color scale indicates the contact frequency between chromosome regions. Blue indicates low contact frequency and red indicates high contact frequency. **(B)** Chromosome interaction frequency and genomic distance. **(C)** Scalogram representation. Scalograms reflect the relative compactness of the contact distribution of chromosome regions. The colored areas above each bin represent the fraction of the total cumulated contacts made by the bin with flanking regions of increasing sizes (dark blue, 0–15%; light blue, 15–30%, etc.; red, 75–100%). Constrained regions display small blue and large red areas. Loose regions display large blue and small red areas.

Hi-C experiments were performed to explore differences in chromosomal conformations between the control strain and subpopulations M1 and M2 obtained by continuous incubation in SMG. Chromosome interaction heat maps were drawn at 2 kb resolution to identify different chromosome interactions at the genome-wide level. Compared to WT, the chromosome interaction patterns of M1 and M2 were altered (Figure 1A). Under SMG, short-range interactions decreased while long-range interactions increased, indicating that the chromosome folding and compact of M1 and M2 had become looser. To confirm this phenomenon, we analyzed the relationship between chromatin interaction intensity and one-dimensional sequence distance. Compared to WT, M1, and M2 had significantly fewer proximity interactions and more distant interactions, while the negative correlation between the strength of chromosome interactions and distance was diminished (Figure 1B). Referring to a previous study (Lioy et al., 2018), we plotted a scalogram to illustrate the cumulative amount of contact between each bin and its flanking region, reflecting the relative tightness of the contact distribution. Taken together, all the results showed that chromosomes in M1 and M2 were looser compared to the WT (Figure 1C). In addition, 3D simulation of chromosomes showed the same results (Supplementary Figure 1).

### Simulated Microgravity Alters Chromosomal Interaction Regions

**FIGURE 2:**
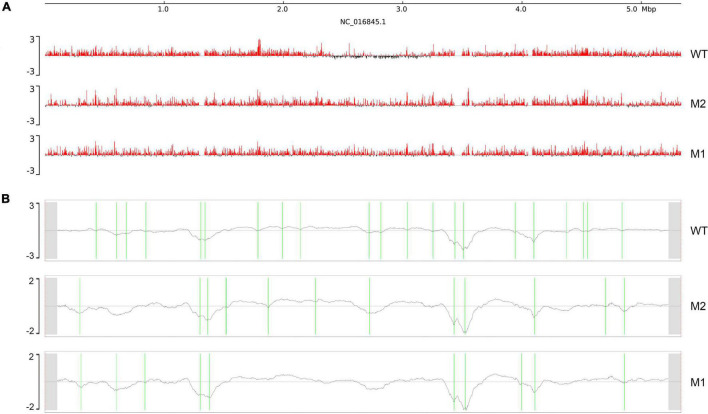
Chromosome interaction structures. **(A)** Chromosome A/B compartments. The x-axis represents genomic coordinates. The y-axis represents the eigenvector values, with positive values in red representing compartment **(A)**, and negative values in black representing compartment **(B)**. **(B)** Chromosomal interaction domains. The x-axis represents genomic coordinates and y-axis represents the insulation index. Green lines indicate CIDs boundaries.

Next, we considered whether there was a corresponding increase in the open chromosome regions and changes in chromosome interactions when chromosome compact structures became looser. Chromosomal domains generally tend to be divided into distinct compartments, labeled “A” and “B.” The more open regions where gene expression is active are defined as A compartments, and the opposite expression-inactive regions are defined as B compartments (Takemata et al., 2019; Misteli, 2020). Therefore, we analyzed the A/B compartments along the chromosome of *K. pneumoniae*. The results showed that compartment A increased and compartment B decreased in M1 and M2, indicating that the active region of the chromosome increased after SMG culture (Figure 2A). In addition, a clear shift from B compartment to A compartment occurred in the replication terminus (ter) region found around position 2.4–3.2 M of the genome. This was consistent with our observation (Figure 1B) that the compacted region in the WT disappeared in M1 and M2. Next, we used the index of insulation score to delineate the CIDs and identified 21, 13, and 10 potential CIDs in WT, M2, and M1, respectively (Table 1). The CIDs were relatively reduced after SMG, and several originally smaller CIDs observed in the WT merged to form a larger CID in M1 and M2 (Figure 2B). These structural changes are closely related to gene expression, which will be analyzed in later sections.

**TABLE 1:** Boundaries location of CIDs.

WT-CID boundaries	Position		M2-CID boundaries	Position		M1-CID boundaries	Position	
ID_1	428,001	430,000	ID_1	292,001	294,000	ID_1	302,001	304,000
ID_2	600,001	602,000	ID_2	1,300,001	1,302,000	ID_2	598,001	600,000
ID_3	682,001	684,000	ID_3	1,364,001	1,366,000	ID_3	838,001	840,000
ID_4	846,001	848,000	ID_4	1,518,001	1,520,000	ID_4	1,302,001	1,304,000
ID_5	1,306,001	1,308,000	ID_5	1,520,001	1,522,000	ID_5	1,378,001	1,380,000
ID_6	1,342,001	1,344,000	ID_6	1872001	1,874,000	ID_6	3,430,001	3,432,000
ID_7	1786001	1,788,000	ID_7	2,268,001	2,270,000	ID_7	3,524,001	3,526,000
ID_8	1,990,001	1,992,000	ID_8	2,722,001	2,724,000	ID_8	3,996,001	3,998,000
ID_9	2,142,001	2,144,000	ID_9	3,430,001	3,432,000	ID_9	4,108,001	4,110,000
ID_10	2718001	2,720,000	ID_10	3,522,001	3,524,000	ID_10	4,858,001	4,860,000
ID_11	2816001	2,818,000	ID_11	4,106,001	4,108,000			
ID_12	3,038,001	3,040,000	ID_12	4,700,001	4,702,000			
ID_13	3,254,001	3,256,000	ID_13	4,858,001	4,860,000			
ID_14	3,438,001	3,440,000						
ID_15	3510001	3,512,000						
ID_16	3,944,001	3,946,000						
ID_17	4,100,001	4,102,000						
ID_18	4,374,001	4,376,000						
ID_19	4,516,001	4,518,000						
ID_20	4,550,001	4,552,000						
ID_21	4,840,001	4,842,000						

### Simulated Microgravity Strongly Influenced Membrane-Related Genes’ Expression

**FIGURE 3:**
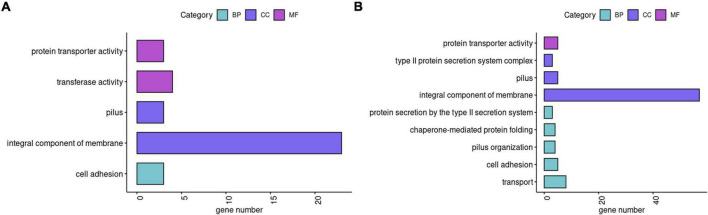
Functions of DEGs. **(A,B)** Classification of up-regulated genes in M1 and M2 compared with WT, respectively. In GO enrichment analysis, BP, CC, and MF are the categories of Gene Ontology, representing biological processes, cellular components, and molecular functions, respectively.

To investigate the effect of SMG on *K. pneumoniae* gene expression, we performed transcriptome analysis. Using a twofold threshold, M1 and M2 had 215 and 304 DEGs, respectively, compared to the WT (Supplementary Tables 1, 2). Although parts of the genes had not been annotated by GO or KEGG databases, for M1 we still found GO and KEGG annotations of 23 upregulated genes related to the integral component of membrane and further upregulation of the genes involved in ribosome, protein transporter activity, transferase activity, cell adhesion, and pilus (Figure 3A). Downregulated genes in the M1 strain were those involved in transporter activity and cell adhesion (Supplementary Table 1). In M2, 57 upregulated genes were related to the integral components of the membrane and other upregulated genes were mainly involved in transport, cell adhesion, and pilus (Figure 3B). Downregulated genes in M2 were also related to integral components of the membrane (Supplementary Table 2).

In addition, cellulose synthesis-related genes (KPHS_03110, KPHS_03880, KPHS_03890, KPHS_43460, and KPHS_43590), as well as biofilm formation-related glycosyltransferase genes (KPHS_03620, KPHS_28150, and KPHS_30480) were upregulated in M2. These findings are consistent with previous studies showing that M2 has a high capacity for biofilm formation, with the biofilm being mainly composed of cellulose (Wang et al., 2017). The expression of five genes (KPHS_28260, KPHS_28270, KPHS_30010, KPHS_30020, and KPHS_30030) associated with quorum sensing, one of the important regulatory mechanisms of bacterial biofilm formation (Solano et al., 2014), was also upregulated in M2 (Table 2).

**TABLE 2:** DEGs associated with biofilm formation in M2.

Gene ID	Position		Product	Differential expression
KPHS_03110	350,335	350,892	Putative fimbrial chaperone protein	UP (log2Foldchange = 1.19)
KPHS_03620	397,064	398,392	Biofilm PGA synthesis N-glycosyltransferase PgaC	UP (log2Foldchange = 1.24)
KPHS_03880	427,295	427,981	Fimbrial biogenesis periplasmic chaperone	UP (log2Foldchange = 1.89)
KPHS_03890	428,039	428,602	Type 1 fimbrial protein	UP (log2Foldchange = 1.53)
KPHS_28150	2,816,588	2,817,283	Putative glycosyltransferase	UP (log2Foldchange = 6.63)
KPHS_28260	2,825,812	2,826,660	Putative ABC transport system permease	UP (log2Foldchange = 1.28)
KPHS_28270	2,826,657	2,828,273	ABC superfamily ATP binding cassette transporter, ABC protein	UP (log2Foldchange = 1.79)
KPHS_30010	2,991,525	2,992,484	Oligopeptide ABC transport system permease component	UP (log2Foldchange = 3.72)
KPHS_30020	2,992,481	2,993,431	Oligopeptide ABC transport system permease component	UP (log2Foldchange = 1.07)
KPHS_30030	2,993,428	2,994,408	Putative ABC transport system ATP-binding protein	UP (log2Foldchange = 1.93)
KPHS_30480	3,037,547	3,038,482	Glycosyltransferase	UP (log2Foldchange = 2.15)
KPHS_43460	4,383,912	4,384,637	MrkB fimbrial protein	UP (log2Foldchange = 1.01)
KPHS_43590	4,396,756	4,397,283	Type 1 fimbrial minor component	UP (log2Foldchange = 1.26)

### Differentially Expressed Genes Were Enriched in Changeable Chromosomal Interaction Domains Boundaries

The spatial organization of the genome is essential for modulating gene regulation (Kong and Zhang, 2019). In mammalian T cells, there is a non-random overlap between regions of chromosome structural changes and regions of DEGs in response to gravity changes (Vahlensieck et al., 2021). In addition, our previous study found that under SMG, most DEGs of *Vibrio natriegens* were located in regions with changeable chromosome structures (Yin et al., 2020).

**FIGURE 4:**
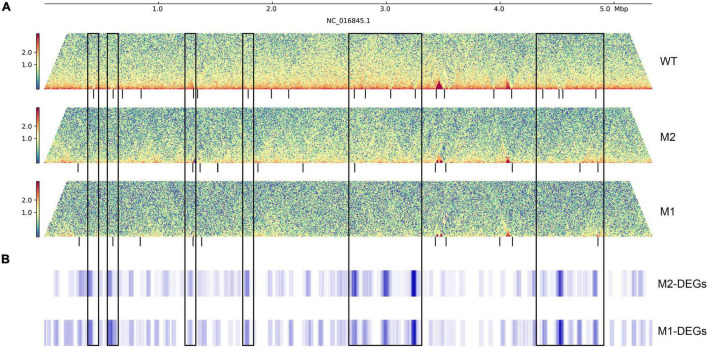
The distribution of DEGs on the genome. **(A)** Interaction heat map of chromosomes, the black vertical line below represents CID boundaries. The color scale indicates the contact frequency between chromosome regions. Blue indicates low contact frequency and red indicates high contact frequency. **(B)** Distribution of DEGs in the genome. The black box represents the CID boundaries with DEGs enrichment.

To further investigate the relationship between DEGs and chromosome structure in *K. pneumoniae* under SMG, we analyzed the distribution of DEGs on chromosomes using a 40 kb window and a 2 kb sliding step. DEGs were significantly enriched near some changeable CID boundaries, with more than twice the enrichment fold than their distribution at the genome-wide level (Figures 4A,B and Supplementary Figure 2). These variable CID boundaries were categorized into two main types: the first was CID boundaries present in WT but disappearing in M1 or M2 (CID boundaries ID-1,2,7,10,11,12,13,18,19,20 of WT), and the second was relatively conserved boundaries in all three strains (CID boundaries ID-5,21 of WT). DEGs were enriched in these regions and were mainly upregulated, and some of them were involved in physiological activities related to membrane components, pili, and phagosomes, while others were mostly putative genes of unknown function.

### Reduced Methylation Levels in the Genome

Several studies have shown that DNA methylation plays an important role in regulating gene expression during space flight (Singh et al., 2010; Kamal et al., 2018; Lei et al., 2020). We analyzed the methylation levels of WT, M1, and M2, and counted their differentially methylated regions (DMRs). Overall, the M1 and M2 genomes were in a hypomethylated state (Supplementary Figure 3 and Supplementary Tables 3, 4). However, we did not observe a significant association between these regions and the DEGs. In addition, although methylation levels are reduced near some regions with boundary changes, whether they are related to domain formation remains to be explored.

## Discussion

In bacteria, the three-dimensional higher-order structure of chromosomes is one of the important ways of epigenetic regulation and plays a very important role in gene transcription, chromosome replication and division. Under SMG, we had screened a mutant of *Vibrio natriegens* with slower growth rate. The significant number of structure variations and the decreased interaction frequency in regions associated with well-orchestrated chromosomes replication may be the main reasons for the slower growth rate of *V. natriegens* (Yin et al., 2020). Though *Klebsiella pneumoniae* exhibited phenotypically heterogeneous growth characteristics under SMG, whole genome sequencing did not reveal significant single nucleotide changes (Wang et al., 2017). These suggest that different strains may have different mechanisms for coping with microgravity. In this study, we investigated the chromosomal conformation of *K. pneumoniae* and its subpopulations M1 and M2 obtained after 2 weeks of continuous SMG incubation and explored the potential relationships between genome organization, transcriptome and genome methylation. Overall, under SMG, the chromosome structure became looser and more exposed, most DEGs were upregulated, and the corresponding methylation level was reduced. Although we did not observe a strong association between methylation and gene expression, DEGs were significantly enriched near the CID boundaries, suggesting that chromosome structure plays a dominant role in the regulation of gene expression, at least in our SMG conditions.

Genome-wide chromosome conformation capture (Hi-C) analysis of M1 and M2 revealed that the chromosome structures after incubation in an SMG environment were significantly different compared to WT, and the degree of chromosome looseness in M1 and M2 was also much higher than WT as determined form 3D simulation results. A/B compartment analysis also revealed that chromosomes became more active. For eukaryotes, Vahlensieck et al. (2021) reported that under normal gravity the nucleus is compressed by the force of gravity along the vertical axis, thus shortening it along the vertical axis and elongating it along the horizontal axis. In microgravity, these forces are no longer present, and the shape of the nucleus becomes more circular (Vahlensieck et al., 2021). In prokaryotes, similar change may occur in the microorganism chromatin, as suggested by our present and previous results.

With the merging and shifting of CIDs, the CIDs in M1 and M2 were reduced and transformed from small to large domains. Related studies in *C. crescentus* have found that long and active transcriptional regions drive the establishment of chromosomal structures and reduce the mutual contact between neighboring structures (Le and Laub, 2016). We found that DEGs were significantly enriched near CID boundaries and were mostly upregulated in M1 and M2. This might be caused by the disappearance of some boundaries in M1 and M2, leading to a higher frequency of contact between these genes and neighboring genes, driving an upregulation of their gene expression. There might be other specific factors involved in CID boundary formation and this warrants further investigation.

Under SMG, we observed that the replication terminus (ter) region of *K. pneumoniae* was changed in terms of both the structure of the chromosome and A/B compartments (Figures 1C, 2A). The ter region can be further divided into four CID structures, which may play an important role in the formation of the ter region because the state of the ter region is consistent with the rest of the chromosome in M1 and M2. This is probably because the fusion of the originally existing four CID regions occurred, and the disappearance of the boundaries led to transformation of the ter region, which also led to differential expression of a number of genes. In addition, we observed a general decrease in methylation levels at the regions of where these four CID boundaries were previously present (Supplementary Figure 3), suggesting a relationship between the domains and methylation.

The membrane fluidity of bacteria is significantly altered in microgravity environments, leading to changes in activity and heat tolerance (Vukanti et al., 2012; Kim and Rhee, 2016). Our results revealed that a large number of genes related to membrane structure were affected by the effects of microgravity; however, the specific biological mechanisms remain to be investigated. In addition, bacterial biofilm formation is closely related to the environment and can change in response to variable environmental (O’Toole et al., 2000). Bacterial biofilms have been explored in several microgravity studies, and it was found that thicker biofilms are formed under these conditions compared to normal gravity, which may increase antibiotic resistance and affect bacterial community aggregation behavior (Tucker et al., 2007; Searles et al., 2011; Kim et al., 2013). Quorum sensing regulates biofilm formation (Costerton et al., 2005; Solano et al., 2014) and cellulose is the main component of biofilm formation. We found that quorum sensing, cellulose biosynthesis, and glycosyltransferase-related genes were significantly upregulated in M2, which may explain the increased biofilm formation for M2 and might also be related to the formation of the beaded chain-like aggregation behavior of M2 (Wang et al., 2017). These genes were located at changeable CID boundaries, suggesting that chromosome structure affects gene expression and thus alters the phenotype. However, more evidence is needed to prove this, and it will be interesting to continue exploring this issue.

## Data Availability Statement

The datasets presented in this study can be found in online repositories. The names of the repository/repositories and accession number(s) can be found below: NCBI BioProject—PRJNA809422.

## Author Contributions

YW and WS contributed to the experimental operation, data analysis, and manuscript draft writing. BY, SS, GB, XG, and KL contributed to the experimental operation. MY, WH, PL, YFJ, and YZ contributed to the data analysis. WS, YQJ, JW, YH, and ZZ contributed to the experimental design, supervision, and manuscript editing. All authors reviewed, revised, and approved the final report.

## Conflict of Interest

The authors declare that the research was conducted in the absence of any commercial or financial relationships that could be construed as a potential conflict of interest.

## Publisher’s Note

All claims expressed in this article are solely those of the authors and do not necessarily represent those of their affiliated organizations, or those of the publisher, the editors and the reviewers. Any product that may be evaluated in this article, or claim that may be made by its manufacturer, is not guaranteed or endorsed by the publisher.

## Supplementary Material

Supplementary Figure 1 3D representation of chromosome structure. The color gradient shows the genome coordinates, with blue and red indicating the start and end points of the genome, respectively.

Supplementary Figure 2 Enrichment of DEGs at the CID boundaries of WT. x axes represent ID of CIDs, y axes represent log2 value of DEGs enrichment.

Supplementary Figure 3 DMRs of M1 and M2 compared to WT. The x-axis represents genomic coordinates and y-axis represents the values of methylation difference. Red represents up-regulated DMRs, green represents down-regulated DMRs.

## References

[B1] Aunins T. R. Erickson K. E. Prasad N. Levy S. E. Jones A. Shrestha S. (2018). Spaceflight modifies *Escherichia coli* gene expression in response to antibiotic exposure and reveals role of oxidative stress response. *Front. Microbiol.* 9:310. 10.3389/fmicb.2018.00310 29615983 PMC5865062

[B2] Baker P. W. Meyer M. L. Leff L. G. (2004). *Escherichia coli* growth under modeled reduced gravity. *Microgravity Sci. Technol.* 15 39–44. 10.1007/BF02870967 15768486

[B3] Bu D. Luo H. Huo P. Wang Z. Zhang S. He Z. (2021). KOBAS-i: intelligent prioritization and exploratory visualization of biological functions for gene enrichment analysis. *Nucleic Acids Res.* 49 W317–W325. 10.1093/nar/gkab447 34086934 PMC8265193

[B4] Costerton J. W. Montanaro L. Arciola C. R. (2005). Biofilm in implant infections: its production and regulation. *Int. J. Artif. Organs* 28 1062–1068. 10.1177/039139880502801103 16353112

[B5] Crabbé A. Nielsen-Preiss S. M. Woolley C. M. Barrila J. Buchanan K. McCracken J. (2013). Spaceflight enhances cell aggregation and random budding in *Candida albicans*. *PLoS One* 8:e80677. 10.1371/journal.pone.0080677 24324620 PMC3851762

[B6] Ewels P. Magnusson M. Lundin S. Käller M. (2016). MultiQC: summarize analysis results for multiple tools and samples in a single report. *Bioinformatics* 32 3047–3048. 10.1093/bioinformatics/btw354 27312411 PMC5039924

[B7] Huang D. W. Sherman B. T. Lempicki R. A. (2009a). Bioinformatics enrichment tools: paths toward the comprehensive functional analysis of large gene lists. *Nucleic Acids Res.* 37 1–13. 10.1093/nar/gkn923 19033363 PMC2615629

[B8] Huang D. W. Sherman B. T. Lempicki R. A. (2009b). Systematic and integrative analysis of large gene lists using DAVID bioinformatics resources. *Nat. Protoc.* 4 44–57. 10.1038/nprot.2008.211 19131956

[B9] Kamal K. Y. Herranz R. van Loon J. J. W. A. Medina F. J. (2018). Simulated microgravity, Mars gravity, and 2g hypergravity affect cell cycle regulation, ribosome biogenesis, and epigenetics in *Arabidopsis* cell cultures. *Sci. Rep.* 8:6424. 10.1038/s41598-018-24942-7 29686401 PMC5913308

[B10] Kim H. W. Rhee M. S. (2016). Influence of low-shear modeled microgravity on heat resistance, membrane fatty acid composition, and heat stress-related gene expression in *Escherichia coli* O157:H7 ATCC 35150, ATCC 43889, ATCC 43890, and ATCC 43895. *Appl. Environ. Microbiol.* 82 2893–2901. 26944847 10.1128/AEM.00050-16 PMC4959082

[B11] Kim W. Tengra F. K. Shong J. Marchand N. Chan H. K. Young Z. (2013). Effect of spaceflight on *Pseudomonas aeruginosa* final cell density is modulated by nutrient and oxygen availability. *BMC Microbiol.* 13:241. 10.1186/1471-2180-13-241 24192060 PMC4228280

[B12] Kong S. Zhang Y. (2019). Deciphering Hi-C: from 3D genome to function. *Cell Biol. Toxicol.* 35 15–32. 10.1007/s10565-018-09456-2 30610495

[B13] Krueger F. Andrews S. R. (2011). Bismark: a flexible aligner and methylation caller for Bisulfite-Seq applications. *Bioinformatics* 27 1571–1572. 10.1093/bioinformatics/btr167 21493656 PMC3102221

[B14] Lajoie B. Venev S. (2016). *Dekkerlab/cworld-dekker: v1.0.0*. Available online at: https://github.com/dekkerlab/cworld-dekker

[B15] Langmead B. Salzberg S. L. (2012). Fast gapped-read alignment with Bowtie 2. *Nat. Methods* 9 357–359. 10.1038/nmeth.1923 22388286 PMC3322381

[B16] Le T. B. Laub M. T. (2016). Transcription rate and transcript length drive formation of chromosomal interaction domain boundaries. *EMBO J.* 35 1582–1595. 10.15252/embj.201593561 27288403 PMC4946140

[B17] Le T. B. Imakaev M. V. Mirny L. A. Laub M. T. (2013). High-resolution mapping of the spatial organization of a bacterial chromosome. *Science* 342 731–734. 10.1126/science.1242059 24158908 PMC3927313

[B18] Lei X. Cao Y. Ma B. Zhang Y. Ning L. Qian J. (2020). Development of mouse preimplantation embryos in space. *Natl. Sci. Rev.* 7 1437–1446. 10.1093/nsr/nwaa062 34691539 PMC8288510

[B19] Li J. Liu F. Wang Q. Ge P. Woo P. C. Yan J. (2014). Genomic and transcriptomic analysis of NDM-1 *Klebsiella pneumoniae* in spaceflight reveal mechanisms underlying environmental adaptability. *Sci. Rep.* 4:6216. 10.1038/srep06216 25163721 PMC4147364

[B20] Lieberman-Aiden E. van Berkum N. L. Williams L. Imakaev M. Ragoczy T. Telling A. (2009). Comprehensive mapping of long-range interactions reveals folding principles of the human genome. *Science* 326 289–293. 10.1126/science.1181369 19815776 PMC2858594

[B21] Lioy V. S. Cournac A. Marbouty M. Duigou S. Mozziconacci J. Espéli O. (2018). Multiscale structuring of the *E. coli* chromosome by nucleoid-associated and condensin proteins. *Cell* 172 771–783.e18. 10.1016/j.cell.2017.12.027 29358050

[B22] Lourenço R. F. Saurabh S. Herrmann J. Wakatsuki S. Shapiro L. (2020). The nucleoid-associated protein GapR uses conserved structural elements to oligomerize and bind DNA. *mBio* 11:e00448-20. 10.1128/mBio.00448-20 32518183 PMC7373187

[B23] Love M. I. Huber W. Anders S. (2014). Moderated estimation of fold change and dispersion for RNA-seq data with DESeq2. *Genome Biol.* 15:550. 10.1186/s13059-014-0550-8 25516281 PMC4302049

[B24] Lynch S. V. Mukundakrishnan K. Benoit M. R. Ayyaswamy P. S. Matin A. (2006). *Escherichia coli* biofilms formed under low-shear modeled microgravity in a ground-based system. *Appl. Environ. Microbiol.* 72 7701–7710. 10.1128/AEM.01294-06 17028231 PMC1694224

[B25] Marbouty M. Le Gall A. Cattoni D. I. Cournac A. Koh A. Fiche J. B. (2015). Condensin- and replication-mediated bacterial chromosome folding and origin condensation revealed by Hi-C and super-resolution imaging. *Mol. Cell* 59 588–602. 10.1016/j.molcel.2015.07.020 26295962

[B26] Misteli T. (2020). The self-organizing genome: principles of genome architecture and function. *Cell* 183 28–45. 10.1016/j.cell.2020.09.014 32976797 PMC7541718

[B27] Mohd Asri N. A. Ahmad S. Mohamud R. Mohd Hanafi N. Mohd Zaidi N. F. Irekeola A. A. (2021). Global prevalence of nosocomial multidrug-resistant *Klebsiella pneumoniae*: a systematic review and meta-analysis. *Antibiotics (Basel)* 10:1508. 10.3390/antibiotics10121508 34943720 PMC8698758

[B28] Novikova N. D. (2004). Review of the knowledge of microbial contamination of the Russian manned spacecraft. *Microb. Ecol.* 47 127–132. 10.1007/s00248-003-1055-2 14994178

[B29] O’Toole G. Kaplan H. B. Kolter R. (2000). Biofilm formation as microbial development. *Annu. Rev. Microbiol.* 54 49–79. 10.1146/annurev.micro.54.1.49 11018124

[B30] Qin L. Erkelens A. M. Ben Bdira F. Dame R. T. (2019). The architects of bacterial DNA bridges: a structurally and functionally conserved family of proteins. *Open Biol.* 9:190223. 10.1098/rsob.190223 31795918 PMC6936261

[B31] Searles S. C. Woolley C. M. Petersen R. A. Hyman L. E. Nielsen-Preiss S. M. (2011). Modeled microgravity increases filamentation, biofilm formation, phenotypic switching, and antimicrobial resistance in *Candida albicans*. *Astrobiology* 11 825–836. 10.1089/ast.2011.0664 21936634

[B32] Singh K. P. Kumari R. Dumond J. W. (2010). Simulated microgravity-induced epigenetic changes in human lymphocytes. *J. Cell. Biochem.* 111 123–129. 10.1002/jcb.22674 20506542

[B33] Solano C. Echeverz M. Lasa I. (2014). Biofilm dispersion and quorum sensing. *Curr. Opin. Microbiol.* 18 96–104. 10.1016/j.mib.2014.02.008 24657330

[B34] Su X. Guo Y. Fang T. Jiang X. Wang D. Li D. (2021). Effects of simulated microgravity on the physiology of *Stenotrophomonas maltophilia* and multiomic analysis. *Front. Microbiol.* 12:701265. 10.3389/fmicb.2021.701265 34512577 PMC8429793

[B35] Takemata N. Samson R. Y. Bell S. D. (2019). Physical and functional compartmentalization of archaeal chromosomes. *Cell* 179 165–179.e18. 10.1016/j.cell.2019.08.036 31539494 PMC6756186

[B36] Tucker D. L. Ott C. M. Huff S. Fofanov Y. Pierson D. L. Willson R. C. (2007). Characterization of *Escherichia coli* MG1655 grown in a low-shear modeled microgravity environment. *BMC Microbiol.* 7:15. 10.1186/1471-2180-7-15 17343762 PMC1852313

[B37] Vahlensieck C. Thiel C. S. Zhang Y. Huge A. Ullrich O. (2021). Gravitational force-induced 3D chromosomal conformational changes are associated with rapid transcriptional response in human T cells. *Int. J. Mol. Sci.* 22:9426. 10.3390/ijms22179426 34502336 PMC8430767

[B38] Venev S. Abdennur N. Goloborodko A. Flyamer I. Fudenberg G. Nuebler J. (2021). *mirnylab/cooltools: v0.4.1*. 10.5281/zenodo.5214125

[B39] Verma S. C. Qian Z. Adhya S. L. (2019). Architecture of the *Escherichia coli* nucleoid. *PLoS Genet.* 15:e1008456. 10.1371/journal.pgen.1008456 31830036 PMC6907758

[B40] Vukanti R. Mintz E. Leff L. (2008). Changes in gene expression of *E. coli* under conditions of modeled reduced gravity. *Microgravity Sci. Technol.* 20 41–57. 10.1007/s12217-008-9012-9

[B41] Vukanti R. Model M. A. Leff L. G. (2012). Effect of modeled reduced gravity conditions on bacterial morphology and physiology. *BMC Microbiol.* 12:4. 10.1186/1471-2180-12-4 22239851 PMC3274431

[B42] Wang H. Li W. Gu L. Gao X. Ni B. Deng H. (2017). Emergence of two distinct subpopulations from *Klebsiella pneumoniae* grown in the stimulated microgravity environment. *Future Microbiol.* 12 939–951. 10.2217/fmb-2017-0032 28816520

[B43] Wang H. Yan Y. Rong D. Wang J. Wang H. Liu Z. (2016). Increased biofilm formation ability in *Klebsiella pneumoniae* after short-term exposure to a simulated microgravity environment. *Microbiologyopen* 5 793–801. 10.1002/mbo3.370 27185296 PMC5061716

[B44] Wang X. Le T. B. Lajoie B. R. Dekker J. Laub M. T. Rudner D. Z. (2015). Condensin promotes the juxtaposition of DNA flanking its loading site in *Bacillus subtilis*. *Genes Dev.* 29 1661–1675. 10.1101/gad.265876.115 26253537 PMC4536313

[B45] Wang X. Montero Llopis P. Rudner D. Z. (2013). Organization and segregation of bacterial chromosomes. *Nat. Rev. Genet.* 14 191–203. 10.1038/nrg3375 23400100 PMC3869393

[B46] Wolff J. Rabbani L. Gilsbach R. Richard G. Manke T. Backofen R. (2020). Galaxy HiCExplorer 3: a web server for reproducible Hi-C, capture Hi-C and single-cell Hi-C data analysis, quality control and visualization. *Nucleic Acids Res.* 48 W177–W184. 10.1093/nar/gkaa220 32301980 PMC7319437

[B47] Wu H. Xu T. Feng H. Chen L. Li B. Yao B. (2015). Detection of differentially methylated regions from whole-genome bisulfite sequencing data without replicates. *Nucleic Acids Res.* 43:e141. 10.1093/nar/gkv715 26184873 PMC4666378

[B48] Yin M. Ye B. Jin Y. Liu L. Zhang Y. Li P. (2020). Changes in *Vibrio natriegens* growth under simulated microgravity. *Front. Microbiol.* 11:2040. 10.3389/fmicb.2020.02040 32983034 PMC7483581

[B49] Zea L. Larsen M. Estante F. Qvortrup K. Moeller R. Dias de Oliveira S. (2017). Phenotypic changes exhibited by *E. coli* cultured in space. *Front. Microbiol.* 8:1598. 10.3389/fmicb.2017.01598 28894439 PMC5581483

