# Supplementary figures and images for: Changes in Higher-Order Chromosomal Structure of Klebsiella pneumoniae Under Simulated Microgravity
Source: Front Microbiol. 2022 May 30;13:879321. doi: 10.3389/fmicb.2022.879321 (PMC9197264; doi:10.3389/fmicb.2022.879321)

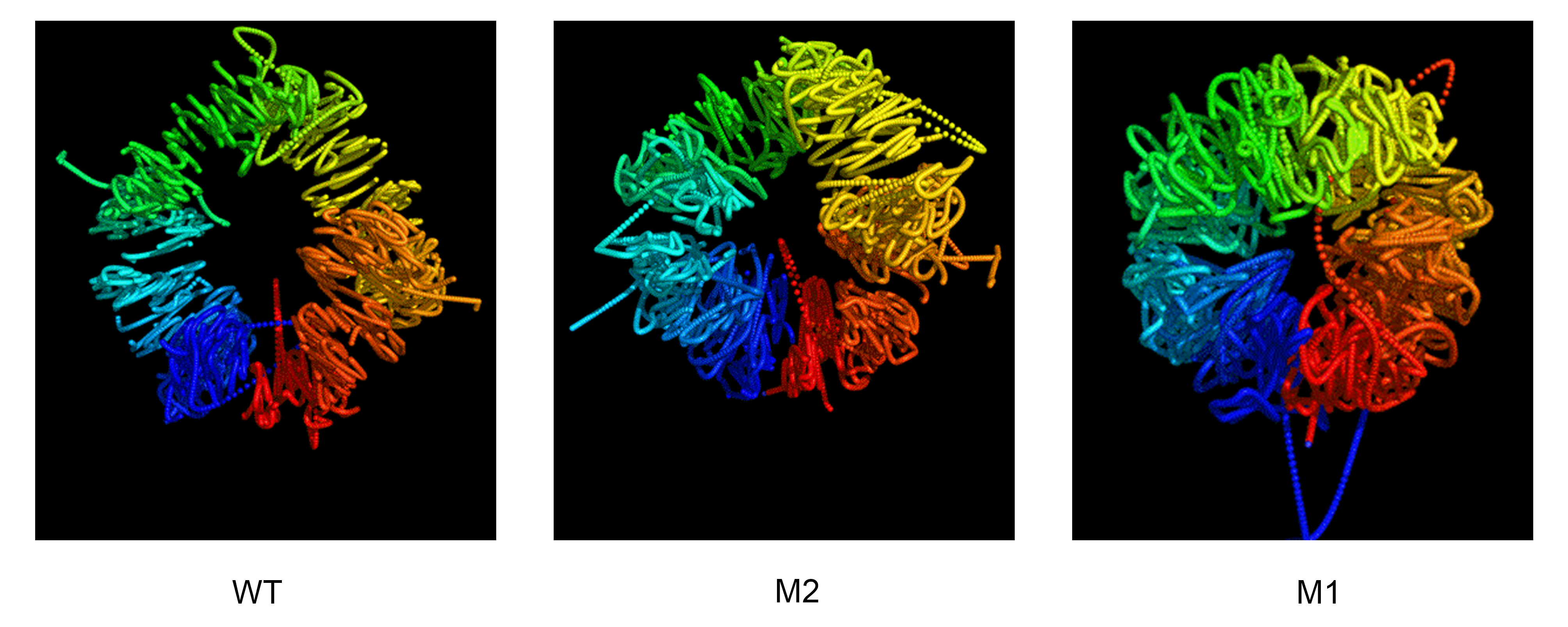

Supplement: Supplementary Figure 1 — 3D representation of chromosome structure. The color gradient shows the genome coordinates, with blue and red indicating the start and end points of the genome, respectively. [file Image_1.JPEG]

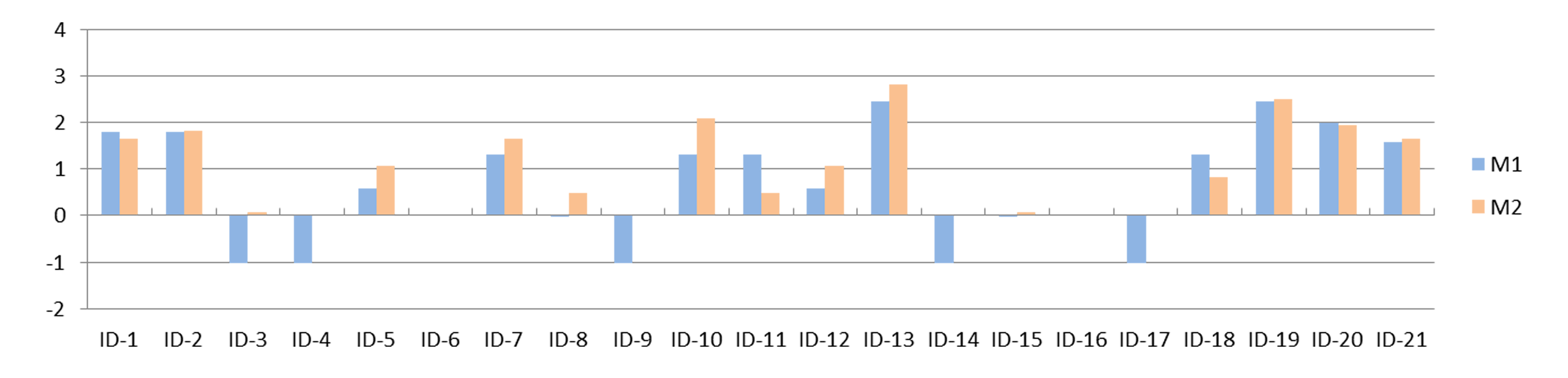

Supplement: Supplementary Figure 2 — Enrichment of DEGs at the CID boundaries of WT. x axes represent ID of CIDs, y axes represent log2 value of DEGs enrichment. [file Image_2.JPEG]

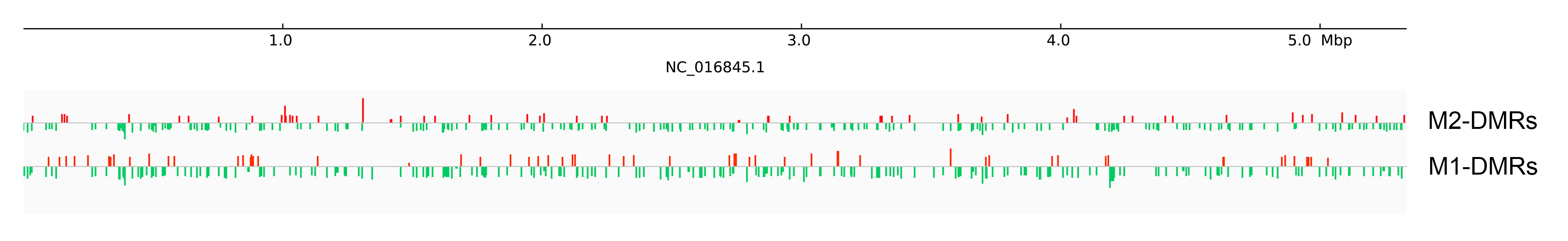

Supplement: Supplementary Figure 3 — DMRs of M1 and M2 compared to WT. The x-axis represents genomic coordinates and y-axis represents the values of methylation difference. Red represents up-regulated DMRs, green represents down-regulated DMRs. [file Image_3.JPEG]
